# Regarding the importance of population diversity validation in Alzheimer's disease models

**DOI:** 10.1002/alz.71071

**Published:** 2026-01-14

**Authors:** Paul D. Coleman, Elaine Delvaux, Ashley Boehringer, Carol J. Huseby

**Affiliations:** ^1^ Arizona State University Banner‐Neurodegenerative Disease Research Center Biodesign Institute Tempe Arizona USA

**Keywords:** Alzheimer's disease, cellular stress response, population diversity, stress granules

1

In our review article titled “Massive changes in gene expression and their cause(s) can be a unifying principle in the pathobiology of Alzheimer's disease,”^1^ we posed an answer to the question, “What mechanisms might be responsible for the massive changes in gene expression encompassing over 90% of the known KEGG pathways?” ^2^, ^3^ This question has confounded the neurodegenerative disease field for years, complicating efforts focusing on single pathways for the development of therapies to slow or halt the progression of Alzheimer's disease (AD). A model that begins with cellular stress response (CSR) offers a unifying mechanism, which when chronic, results in massive gene expression changes in most functional pathways.

CSR occurs within all cells, from simple bacteria to highly differentiated animal cells, when encountering perturbations to homeostatic conditions either through extracellular or intracellular stressors. The CSR program is evolutionarily conserved and reacts to counteract insults, cope, and repair damage. Stress granules (SGs) are a central contributor to the response to shut down macromolecular synthesis and focus on increasing synthesis of stress proteins to mitigate and repair damage. Once indications of the cell stress are gone, the SGs are disassembled via autophagic vesicles and fusion with lysosomes for breakdown^4^ so the cell can return to homeostasis. If survival strategies are unsuccessful, the CSR can promote programmed cell death to eliminate damaged cells.^5^, ^6^

CSRs are conserved across all types of cells with a universal response having dynamic and flexible complexity to offer compensatory mechanisms. The universality of the response comes about through a system of stress monitoring that is based on the damage, thus signaling the appropriate response.^7^ This important aspect of the system, although conserved, can in theory be affected by variations in the molecules involved in the sensing, shift in cellular priorities, and repair of affected molecules.

Dr. Rudroff has outlined evidence for population differences in a letter titled “Population diversity validation for Alzheimer's disease ‘unifying’ models” citing research for genes having significant allele frequency differences across populations. Under our unifying model, molecules that are sequestered in SGs and those with synthesis paused should not affect CSR by population variations; however, those molecules monitoring, initiating, and taking part in damage repair, for example, eIF2α and apolipoprotein E, having variations central to their stress response activity, may enhance or impede the effectiveness of the CSR.^8^ Our model, although having substantial supportive evidence, remains incomplete. Further research is needed to validate multiple aspects of the model. Dr. Rudroff has graciously outlined steps to ensure that validation includes the world's population: (1) multi‐ancestry genetic studies looking for consistency across or within populations; (2) assessment of how environmental exposures of differing populations change the stress response; (3) biomarker development validation in diverse populations before clinical trials; and (4) establishment of international collaborations to foster these important validations.

The conclusion of our work presents a “framework” with multiple areas in need of further investigation. Dr. Rudroff's letter adds an important nuance to our proposed framework and an important lens which we hope future investigations into our proposal will take into consideration. All scientific work benefits from the investigation and inclusion of diverse groups, and while we agree that there may be differing CSRs within individuals and diverse populations, we believe that our hypothesis that CSR is central to the dysfunction in AD and lays the foundation for the massive gene expression changes will remain true.

## CONFLICT OF INTEREST STATEMENT

Carol Huseby, Elaine Delvaux, Paul Coleman, and Ashley Boehringer have nothing to disclose related to the content of this reply. Author disclosures are available in the supporting information.

## Supporting information



Supporting Information
